# Biological and trophic consequences of genetic introgression between endemic and invasive *Barbus* fishes

**DOI:** 10.1007/s10530-021-02577-6

**Published:** 2021-05-26

**Authors:** Vanessa De Santis, Silvia Quadroni, Robert J. Britton, Antonella Carosi, Catherine Gutmann Roberts, Massimo Lorenzoni, Giuseppe Crosa, Serena Zaccara

**Affiliations:** 1 Department of Theoretical and Applied Sciences, University of Insubria, Varese, VA Italy; 2 Life and Environmental Sciences, Bournemouth University, Poole, Dorset UK; 3 Department of Chemistry, Biology and Biotechnologies, University of Perugia, Perugia, PG Italy

**Keywords:** Trophic impacts, *B. plebejus*, *B. tyberinus*, *B. barbus*, Interspecific hybridization, Hybrid vigour

## Abstract

Genetic introgression with native species is recognized as a detrimental impact resulting from biological invasions involving taxonomically similar invaders. Whilst the underlying genetic mechanisms are increasingly understood, the ecological consequences of introgression are relatively less studied, despite their utility for increasing knowledge on how invasion impacts can manifest. Here, the ecological consequences of genetic introgression from an invasive congener were tested using the endemic barbel populations of central Italy, where the invader was the European barbel *Barbus barbus*. Four populations of native *Barbus* species (*B. plebejus* and *B. tyberinus*) were studied: two purebred and two completely introgressed with alien *B. barbus.* Across the four populations, differences in their biological traits (growth, body condition and population demographic structure) and trophic ecology (gut content analysis and stable isotope analysis) were tested. While all populations had similar body condition and were dominated by fish up to 2 years of age, the introgressed fish had substantially greater lengths at the same age, with maximum lengths 410–460 mm in hybrids versus 340–360 mm in native purebred barbel. The population characterized by the highest number of introgressed *B. barbus* alleles (81 %) had the largest trophic niche and a substantially lower trophic position than the other populations through its exploitation of a wider range of resources (e.g. small fishes and plants). These results attest that the genetic introgression of an invasive congener with native species can result in substantial ecological consequences, including the potential for cascading effects.

**Supplementary Information:**

The online version contains supplementary material available at 10.1007/s10530-021-02577-6.

## Introduction

Interspecific hybridization is a widespread process in animal communities that has been suggested to negatively affect species through depressing the fitness of hybrids (i.e. outbreeding depression) (Rhymer and Simberloff 1996). However, growing evidence now suggests that hybridization has driven speciation in a wide range of taxa (Seehausen 2004; Baack and Rieseberg 2007; Svardal et al. 2019) and, consequently, its role in evolution has been reconsidered.

While being an important evolutionary force, introgressive hybridization can create considerable conservation issues (Allendorf et al. 2001; Brennan et al. 2014), especially when anthropogenic activities, such as habitat modification (e.g. Chafin et al. 2019) and species introductions (e.g. Ward et al. 2012), result in the mixing of previously isolated species. This is particularly true when one of the formerly isolated species is an endemic with a narrow distribution range and/or the two species are taxonomically similar (Huxel 1999; Hänfling et al. 2005). Hybridization can even trigger the invasion process (Hovick and Whitney 2014; Roy et al. 2015), with hybrids potentially outperforming parental taxa through the novel combination of parental traits (Seehausen 2004) or expressing new traits through transgressive hybridization (Rieseberg et al. 1999).

Invasion driven hybridization, resulting from the introduction of alien species into communities where taxonomically similar native species are present, is increasingly recognized as a threat to the genetic integrity of many native species (Huxel 1999; Gaskin and Kazmer 2009; Kovach et al. 2015). Current knowledge on the genetic introgression of invasive and native species has tended to focus on the underlying genetic mechanisms, with less consideration given to how the introgression alters the functional traits and ecological interactions of the hybrids in relation to their parental species (Matsuzaki et al. 2010; Toscano et al. 2010; Hayden et al. 2011).

A model to study the ecological consequences of invasive hybridizing species is represented by the European barbel *Barbus barbus* (Linneus 1758), a cyprinid riverine species native to central Europe, that has been introduced into other European areas, including Italy (Bianco and Ketmaier 2001) and Western Britain (Wheeler and Jordan 1990), via anglers or angling orientated stocking events. While this invader has no congeners present in Britain, limiting the genetic introgression concerns (Britton and Pegg 2011), four native *Barbus* species are present in Italy. Two of these, *B. caninus* Bonaparte 1839 and *B. balcanicus* Kotlik, Tsigenopoulus, Rab and Berrebi 2002, inhabit the upper reaches of rivers. In contrast, *B. tyberinus* Bonaparte 1839 and *B. plebejus* Bonaparte 1839, populate the middle/lower reaches of Italian rivers, in habitats that are also preferred by *B. barbus* (Carosi et al. 2017). All these native Italian barbels are generalist benthivores and so their diet tends to be dominated by benthic macroinvertebrates (e.g. dipteran larvae; Tancioni et al. 2001; Piria et al. 2005; Corse et al. 2010), with proportions of other food items varying according to availability (Piria et al. 2005).

Hybridization between *Barbus* species has been widely documented as both natural events (e.g. Tsigenopoulos et al. 2002; Buonerba et al. 2015), and following invasions (Meraner et al. 2013; Geiger et al. 2016). When hybridization occurs in natural contact zones, it is usually limited to that area but, in the case of the genetic admixture between invasive *B. barbus* and native Italian barbels, it has been found to be more widespread, with a tendency to form a complete ‘hybrid swarm’ (e.g. Meraner et al. 2013; Zaccara et al. 2014). Moreover, these *Barbus* hybrids are fertile and a range of hybrid forms may be present across multiple generations, including backcrossed individuals (Meraner et al. 2013). In addition, hybrids between the exotic *B. barbus* and endemic congeneric species are suggested as having enhanced somatic growth rates than their purebred parental species (Meraner et al. 2013; Carosi et al. 2017). There is thus the possibility that hybrid barbel have a fitness at least equal (or higher) to the parental species (Pfennig et al. 2007). Given that this introgression can result in morphological differences between the purebred and hybrid forms (Zaccara et al. 2020), questions have arisen over how morphological shifts alter the interactions of hybrids with other species and their environment, including their utilization of trophic resources.

Therefore, the aim of this study was to test the biological and trophic consequences of genetic introgression across populations of endemic *Barbus* species invaded by *B. barbus*, with comparisons to uninvaded populations. The objectives were to test differences between purebred and introgressed *Barbus* populations in relation to their: (1) somatic growth rate, body condition and population demographic structure (i.e. biological traits); and (2) diet composition and trophic ecology (e.g. trophic niche size and trophic position), enabling the assessment of their functional roles (Davis et al. 2012; De Carvalho et al. 2019; Pacioglu et al. 2019). We posit that: (1) introgressed fish will have biological traits at least equal to those of the parental species; and (2) introgressed fish will have larger trophic niche sizes that differ in their trophic positions compared with native parental species, with this potentially related to alterations in their functional morphology (Zaccara et al. 2020).

## Materials and methods

### Sampling strategy and sites description

**Fig. 1:**
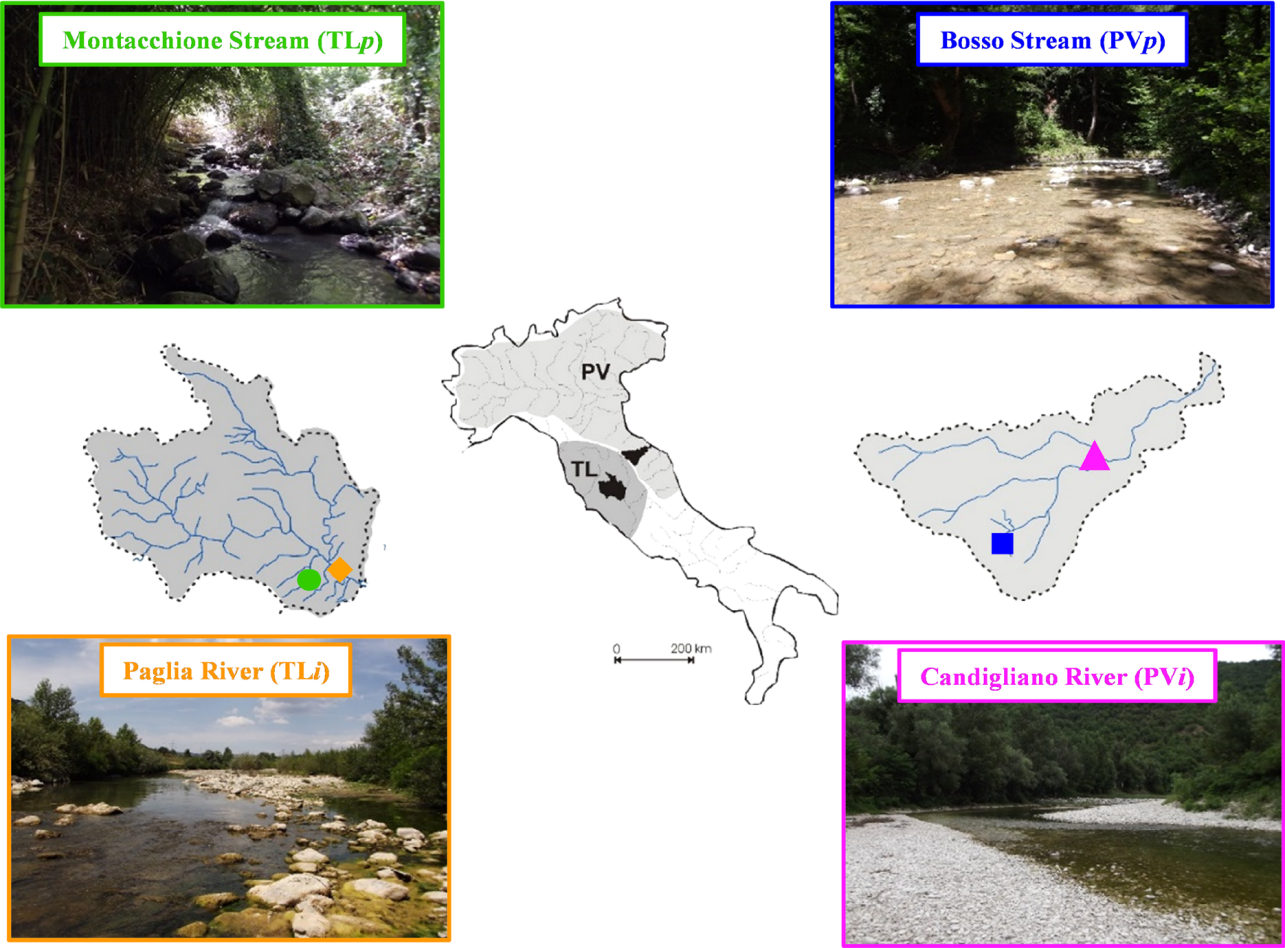
Location and pictures of the four sampled rivers located within the Tuscany-Latium (TL) and the Padano-Venetian districts (PV) where ‘*i*’ and ‘*p*’ indicate sites where hybrid and purebred populations were found. Symbols on maps indicate position of each site where: PV*p =* blue square, PV*i =* pink triangle, TL*p =* green circle and TL*i* = orange diamond

Sampling was performed at four representative sites located in central Italy (Fig. 1; Table 1). Two of these were selected where impassable weirs have prevented *B. barbus* invasions and thus purebred populations of the endemic *B. tyberinus* and *B. plebejus* were present (Zaccara et al. 2020). They were located in the species respective distribution range: the Tuscany-Latium (TL) and the Padano-Venetian (PV) ichthyogeographic districts for *B. tyberinus* and *B. plebejus* respectively (Bianco 1995). The other two sites were located within the same river catchments (see below) but where each of the two native species has introgressed with *B. barbus* (Zaccara et al. 2020) following its invasion of the middle and lower reaches since at least 1998 and 2005 (i.e. their first detections in these basins; Lorenzoni et al. 2010; Lorenzoni and Esposito 2011) in TL and PV districts respectively. Therefore, for each ichthyogeographic district, there was one purebred (“*p*”) population (located in the upstream section), and one invaded and introgressed (“*i*”) population (in the lowland section). Pure vs. hybrid status of populations have already been tested using mitochondrial (D-loop) and nuclear (growth hormone 2; GH-2) DNA markers (see Zaccara et al. 2020). Thus, PV*p* and TL*p* were known to be populated by purebred *B. plebejus* and *B. tyberinus* respectively. Mitochondrial DNA analyses had revealed that barbel in PV*i* and TL*i* were all of hybrid origin (*B. plebejus* × *B. barbus* and *B. tyberinus* × *B. barbus*, respectively), while at the analysed nuclear marker, a different proportion of *B. barbus* alleles was detected between the two invaded populations, resulting in a higher number of *B. barbus* alleles in TL*i* (81 %) than PV*i* (68 %) (Zaccara et al. 2020).

**Table 1:** Habitat characteristics of the four sampling sites and community metrics for macroinvertebrate and fish diversity at each site with relative geographic coordinates, ichthyogeographic district, basin, river and site identification code

Ichthyogeographic district	Padano-Venetian (PV)		Tuscany-Latium (TL)	
Basin	Metauro	Metauro	Tiber	Tiber
River	Bosso	Candigliano	Montacchione	Paglia
Site ID	PV*p*	PV*i*	TL*p*	TL*i*
Geographic coordinates	43°31′3.14"N 12°33′17.89"E	43°38′8.59"N 12°42′41.32"E	42°42′44.39"N 12°5′37.88"E	42°43′38.88"N 12°7′43.00"E
*Habitat characteristics*				
Distance from source (Km)	10	41	10	57
Altitude (m a.m.s.l)	398	201	207	116
River width (m)	7	16	5	17
Canopy cover (%)	90	10	90	10
Geology	Siliceous	Siliceous	Volcanic	Siliceous
*Substrate composition (%)*				
Microlithal (grain size 2–6 cm)	30	60	0	20
Mesolithal (grain size 6–20 cm)	40	40	20	40
Macrolithal (grain size 20–40 cm)	30	0	40	40
Megalithal (grain size > 40 cm)	0	0	40	0
*Mesohabitat typology (%)*				
Riffle	20	30	15	70
Rapid	10	5	10	5
Step	10	5	30	0
Run	40	45	15	10
Pool	20	15	30	5
*Macroinvertebrate community metrics*				
Total density (individuals/m^2^)	1094	5662	1212	848
Family richness	24	19	15	17
Shannon–Wiener diversity (H)	2.0	1.5	1.6	1.5
*Fish community metrics*				
Total density (individuals/m^2^)	3.40	1.97	0.78	8.27
Species richness	6	8	5	8
Shannon–Wiener diversity (H)	0.5	1.4	0.9	0.5

Sites in TL were situated within the Paglia River basin and were the Paglia River (TL*i*) and the Montacchione Stream (TL*p*) (Fig. 1; Table 1), where the latter is a tributary isolated from the main river by the presence of two weirs of over 2 m high. This basin is characterized by impermeable soils, with watercourses flowing in upland areas (Lorenzoni et al. 2010). Sites in PV were located within the Metauro River basin, being the Candigliano River (PV*i*) and the Bosso Stream (PV*p*) (Fig. 1); these sites were separated by three weirs, ranging in height from 0.4 to 1 m. This basin has a mountainous upper section that cuts across an area of steeply folded bedrock (Lorenzoni and Esposito 2011).

All of these watercourses were characterised by marked flow rate oscillations throughout the year and a high susceptibility to drought periods in summer, which are aggravated by water abstraction for irrigation and drinking water supply. The Montacchione sub-basin has a volcanic origin, while the other three are siliceous. Downstream sites (i.e. TL*i* and PV*i*) were characterised by a wider riverbed than the upstream sites, which results in major vegetation cover of the latter that provides shading even during summer droughts. Substrates at all sites were characterised by coarse material, primarily boulders and cobbles in varying percentages, which were mainly mesolithal (6–20 cm) and macrolithal (20–40 cm). Mesohabitats at all sites were mainly characterised by riffle-run hydro-morphologies, except for TL*p* that was dominated by a step-pool hydro-morphology (Table 1; Fig. 1).

### Fish and macroinvertebrate communities sampling and characterisation

Quantitative sampling of the fish communities was completed in July 2019 by electrofishing using DC electric current (2500 W). To estimate fish density, a two-pass electrofishing approach was implemented (Moran 1951; Zippin 1956), involving the survey of a longitudinal transect of length 60 to 112 m (according to river size) in a downstream-upstream direction, applying the same sampling effort twice. Although no stop nets were used, the end of the transects were determined by river morphology (e.g. a significant reduction of riverbed width (i.e. mesohabitat change) or a weir). The sampling was conducted between 10 am and 12 pm in each river; following their capture, the fish were anaesthetised, identified to species where possible (including non-*Barbus* species), measured for total length (to 1 mm) and weight (to 0.1 g).

A quantitative multi-habitat approach (Buffagni et al. 2005) was then used to sample benthic macroinvertebrate (BMI) communities using a Surber sampler (0.1 m^2^ area, 500 μm mesh). Once collected, BMI samples were preserved in formalin (4 %) and, then, in the laboratory, were sorted into families whose density (individuals/m^2^) was determined by counting individuals. For each sampling site, the fish and BMI assemblages were characterised through the calculation of three common metrics: total density (i.e. number of individuals per m^2^), richness (fish: number of species; BMI: number of families) and diversity (Shannon-Wiener index - H; Shannon 1948). The Bray-Curtis index (Bray and Curtis 1957) was used to quantify the compositional dissimilarity between BMI samples, where values ranged from 0 (completely similar) and 1 (completely dissimilar). These analyses were performed with the Past software (Hammer et al. 2001).

### Barbel biological traits

After their measurement, three to five scales were removed from each barbel, taken from the left side, that were used subsequently for age determination. Scalimetry was performed under a stereomicroscope coupled with a camera, with images stored within an archive built with the image analysis system IAS 2000 (QEA’s IASLab® software). Two operators carried out ageing of scales from the images independently, discarding unreadable or dubious scales. Length at age relationships of each population were then fitted to the von Bertalanffy growth model (von Bertalanffy 1938) according to:

$${\text{TL }} = \, {{\text{L}}_\infty }(1 - \exp \, ( - {\text{K }}({\text{t}} - {{\text{t}}_{0}})))$$

Where TL is the total length of each fish in mm at time t, L_∞_ is the theoretical maximum length, K is the rate of approach to the maximum length, and t_0_ is the theoretical age at which TL = 0. To assess possible differences in theoretical growth parameters between populations, different non-linear models were fitted using von Bertalanffy equation in the fisheries assessment R package ‘FSA’ (Ogle et al. 2020; R core team 2019) following a hierarchical approach (Ogle 2013). This consisted in starting with a general model in which L_∞_, K and t_0_ were calculated for each population independently and subsequently simplifying the model by keeping constant initially one and then two parameters at a time, finishing with a model where all the three parameters were in common. The best-fit model was then selected according to the Akaike’s information criterion (AIC; Burnham and Anderson 1998). Differences in length-at-age between populations were tested through MANOVA in R (‘*dplyr*’ package, Wickham et al. 2020), with age and length kept as dependent variables and sampling site retained as independent variable.

Length-weight relations (LWRs) in each population was also estimated using the following linear regression model:

$${\log_{10}}{\text{W }} = {\text{a}} + {\text{b}}{\log_{10}}{\text{TL}}$$

Where W is the weight in grams of the fish, TL is the total length in mm, *a* is the intercept of the regression curve and *b* is the regression coefficient (slope). ANCOVA was used to test for differences in LWR across the populations, with differences from isometric growth (i.e. *b* = 3) tested for each population using *t*-tests. LWR models obtained for each population were then used to back calculate fish weight, and residuals (differences between observed and predicted weight) were tested for significant differences (one way ANOVA) in fish body condition between the populations (Jakob et al. 1996).

### Barbel diet determination through gut content analysis (GCA)

A subsample of barbel (approximately 20 fish per site of age > 1+ and up to 4+, and lengths between 69 and 279 mm) were selected for gut content analysis (GCA). These fish were euthanized (anaesthetic overdose, MS-222), placed on ice and brought to the laboratory. After their defrosting, the fish were dissected, and their guts preserved in ethanol (70 %) prior to analysis. As barbels do not have a differentiated stomach, the entire digestive tract (‘gut’) was weighed and all the prey items examined using microscopy (x 5 to x 50 magnification). Prey were initially identified to the lowest possible taxonomic level before being grouped into 15 categories according to their taxonomic affinities. Food items with a low frequency, low specific abundances (< 5 %) and/or that occurred in only one population, were grouped into broader categories (i.e. terrestrial organisms, other aquatic BMI, fish bones). As the actions of the pharyngeal teeth of barbels makes the separation of their ingested prey difficult, this prevented the effective use of gravimetric or numeric methods and, consequently, the relative-fullness method was selected (Hyslop 1980). This method is recommended as one of the election methods in relative diet composition studies, as it can produce robust data despite its subjective nature (Amundsen and Sánchez-Hernández 2019). Accordingly, gut fullness was estimated on a scale from empty (0 %) to full (100 %), with the volumetric percentage of each food item then estimated by eye and summed up to reach the total fullness. The feeding activity of the fish in each population was then tested comparing the vacuity index (I %; calculated as the proportion of fish with empty stomachs in each sample; Hyslop 1980, Alexandre et al. 2015) and the mean volume of gut contents. To ensure the barbel subsample used for GCA was representative of the diet variation of each fish population, prey accumulation curves were constructed using a sample-based rarefaction method as available in the ‘BiodiversityR’ package in R (Kindt and Coe 2005).

The fish feeding strategy was then assessed following the method proposed by Costello (1990) in its modified version (Amundsen et al. 1996), where the frequency of occurrence and prey-specific abundances of each food category are calculated and used to plot graphs. Visual inspection of the plots indicates prey importance, feeding strategy and, ultimately, how each individual contributes to the trophic niche of the population by specialising on specific dietary items (i.e. within phenotypic contribution) or not (i.e. between phenotypic contribution). Correspondingly, the frequency of occurrence of each food category for each population was calculated as the percentage of fish with prey *i* in their guts against the total number of fish with contents in their guts (*F*_*i*_ % = *N*_*i*_ */ N* x 100). Prey-specific abundances were calculated as the volume occupied by prey item *i* (*S*_*i*_) in all the guts against the total gut volume comprising prey *i* (*P*_*i*_ % = ∑ *S*_*i*_ */* ∑ *S*_*ti *_x 100; Amundsen et al. 1996).

The GCA data were then analysed for diet composition and niche width area per population. Data were arcsine square root transformed and non-metric multidimensional scaling (nMDS) was performed with 40 % standard ellipses representing the core population trophic niche (Gutmann Roberts and Britton 2018), as implemented in the R package ‘*vegan*’ (Oksanen et al. 2019). A Bray-Curtis distance matrix was built before PERMANOVA (‘*adonis*’ function) was used to test for dietary differences between the four populations. SIMPER analysis was then applied to detect the contribution of each food item to the dissimilarities. The Shannon-Wiener diversity index (H) was also calculated within the same package (i.e. *vegan*), and ANOVA and Tukey pairwise test available in R tested for differences in H between the four populations.

### Stable isotope analysis (SIA) of barbel populations and putative food resources

For the stable isotope analyses (SIA), the fish used differed to those used in the GCA but were collected concomitantly. This was partly due to logistical reasons related to both sample collection and to the Covid-19 lockdown that prevented the fish from the GCA being analysed in a timely manner for the purposes of this study. Consequently, 10 barbel per site were analysed for SIA (δ^13^C and δ^15^N). All individuals analysed were of age between 1+ and 4+ years old and of lengths between 98 and 244 mm. Scales were used as the tissue of choice for SIA as they represent non-lethal alternatives to muscles (Busst et al. 2015; Busst and Britton 2018) and are indicative of diet composition over considerable timeframes (> 3 months for *B. barbus*; Busst and Britton 2018). Three to five scales were removed from the left side (above the lateral line and below the dorsal fin) and then placed in paper envelopes until processing.

To provide SIA data for the baselines and putative prey, BMI, biofilm (periphyton), benthic algae and fine and coarse particular organic matter (FPOM and CPOM respectively) were sampled on the same date of the fish. A dedicated BMI sample was collected at each site with the same method used to characterize the BMI communities, but this was put on ice and frozen upon arrival in the laboratory. After defrosting, six families (Baetidae, Chironomidae, Gammaridae (only in TL*p*), Hydropsychidae, Leuctridae (except TL*p*), Simuliidae (except TL*i*)) that were mostly present at all sampling sites were selected (Supplementary material Table S1) and three replicates of each family (comprising of 1 up to 10 individuals, according to the size) were processed. These families mainly comprised a mix of collector gatherers, grazers and scrapers, but also included shredders, filter feeders and predators (www.freshwaterecology.info; Schmidt-Kloiber and Hering 2015). Biofilm was brushed from the upper side of six stones randomly picked up at each sampling site and then combined in 500 ml of water. Samples were frozen until processing in the laboratory, where each sample was divided in three replicates and filtered on glass-fibre filters (0.7 μm pore size). Two litres of turbid water were collected moving fine substrate with hands for FPOM collection, and then three replicates were filtered on glass-fibre filters. CPOM (mainly decaying leaves) and benthic algae (except for TL*p*) were randomly collected by hand at each sampling site and combined to form three replicates.

Preparation for SIA of fish scales, BMI and benthic algae involved rinsing with distilled water before being oven dried at 60 °C for 48 h, with this drying also performed for the biofilm, FPOM and CPOM samples. For the scales, a preliminary step was added that involved the excision of the outer portion of each scale for analysis, as this reflects the collagen produced in the last growth season and not in previous life stages (Hutchinson and Trueman 2006). The stable isotope ratios of carbon (^13^C:^12^C; reported as δ^13^C) and nitrogen (^15^N:^14^N, reported as δ^15^N) of the fish, putative prey and baseline samples were then analysed at the Cornell Isotope Laboratory, New York (USA). Across the four sites, 142 samples were analysed: 40 fish, 57 BMI (five families per site) and 45 primary producers (Supplementary material Table S1). Samples were ground to powder, weighed (to the nearest 1000 µg) and put in tin capsules, before being analysed on a Thermo delta V isotope mass spectrometer (IRMS) coupled with a NC2500 elemental analyser. Data accuracy and precision were tested every 10 samples reporting an overall standard deviation for internal animal standard (deer) of 0.08 for δ^15^N and 0.03 for δ^13^C. The C:N ratios of all animal samples were below 3.5 and so did not require lipid correction (Skinner et al. 2016).

The SIA data were initially tested for any effects of length on δ^13^C and δ^15^N (as a proxy of ontogenetic effects on diet) in each population through linear regressions as implemented in R. A Bayesian approach available in the R package ‘tRophicPosition’ (Quezada-Romegialli et al. 2018) was then implemented to calculate trophic position (TP) at population level and to test for differences in TP between the purebred and hybrid populations. Since the samples for SIA were collected in one season, some of the putative prey, specifically primary producers, may not be reflective of the stable isotope ratios of the fish because of different turnover rates. This, coupled with the fact that barbel are mainly invertivores, led to the use of BMI as the baselines, since they would integrate isotopes on a temporal scale similar to that of the fish (Post et al. 2002). However, as the analysed BMI were not always distinguishable from each other based on their stable isotope ratios (i.e. their standard deviation overlapped; Supplementary material Table S1), then these resources were pooled, resulting in one baseline model being implemented. The trophic discrimination factor used for δ^15^N (i.e. Δ^15^N) was 4.2 ‰ ± 0.2 ‰, with this specific to scales for *B. barbus*, derived experimentally from individuals that had fed on an invertebrate-based diet (Busst and Britton 2016). The probability that the posterior distribution relative to each population’s TP was higher or smaller than others (α = 0.05) was used to test for significant differences.

To enable individual comparisons between the different rivers, barbel δ^15^N ratio was converted to TP according to Olsson et al. (2009):

$${\text{TP }} = \, 2 \, + \, {\delta^{15}}{{\text{N}}_{{\text{barbel}}}} - \, {\delta^{15}}{{\text{N}}_{{\text{meanBMI}}}}/ \, 4.2$$

Where TP and δ^15^N_barbel_ are the trophic position and the nitrogen ratio of each fish and δ^15^N_meanBMI_ is the mean nitrogen ratio of the benthic macroinvertebrates and 2 is the trophic position of this latter (i.e. primary consumers). Although it is recommended to estimate consumer TPs through the use of baseline taxa that are long-lived (e.g. bivalves and snails) (Post 2002), there were insufficient densities of these taxa in the samples to enable this. Similarly, for *Barbus* δ^13^C, conversions to corrected carbon (C_corr_) utilised the δ^13^C data of the BMI using the following equation (adapted from Olsson et al. 2009):

$${{\text{C}}_{{\text{corr}}}} = \, \left[ {\left( {{\delta^{13}}{{\text{C}}_{{\text{barbel}}}} - \, {\Delta^{13}}{\text{C}}} \right) \, - \, {\delta^{13}}{{\text{C}}_{{\text{meanBMI}}}}} \right] \, /{\text{ C}}{{\text{R}}_{{\text{BMI}}}}$$

Wherein δ^13^C_barbel_ is the carbon value of each fish, Δ^13^C is carbon tissue-specific trophic discrimination factor for *B. barbus* fed an invertebrate diet (Busst and Britton 2016), δ^13^C_meanBMI_ is the mean carbon ratio of all the benthic macroinvertebrates sampled for SIA and CR_BMI_ is the carbon range (δ^13^Cmax - δ^13^Cmin) of the same macroinvertebrates (Olsson et al. 2009). ANOVA implemented in R was used to test for differences in carbon source between populations.

The isotopic niches of each population were then built using two approaches in the SIBER R package (Jackson et al. 2011), the maximum likelihood Standard Ellipse Area (SEA) and the Bayesian estimate of the SEA (SEA_B_). SEA_B_ was tested for significant differences in niche width between populations and obtained through Markov Chain Monte Carlo simulations (10^4^ iterations per group), with differences calculated as the probability that the posterior distribution relative to each population niche was larger or smaller than others (α = 0.05). Maximum likelihood estimate of SEAs were used to plot the niches in the isotopic space, where they represent the population ‘core’ niche (40 %), and enabled identification of the extent of isotopic niche overlap between the different barbel populations (Jackson et al. 2012).

## Results

### Characterization of fish and benthic macroinvertebrate communities

The fish communities of the four sites differed considerably in terms of composition, density and richness (Table 1; Supplementary material Table S2). At PV*p*, the dominant species were *Telestes muticellus* (Bonaparte 1837) and *Cottus gobio* Linnaeus 1758; at PV*i*, *Gobio gobio* (Linnaeus 1758) was most abundant; at TL*p* it was *Barbus tyberinus*; and at TL*i* it was *Padogobius nigricans* (Canestrini 1867). These taxa are all native, except for *G. gobio* at PV*i*. At PV*i*, three of eight fish species present were alien and at TL*i*, five of eight were alien (Table S2). All fish species at PV*p* and TL*p* were native, except for two salmonid species (Atlantic lineage of *Salmo trutta* Linnaeus 1758 and *Oncorhyinchus mykiss* Walbaum 1792, respectively). At TL*i* there was the highest density of fishes followed by PV*p*, with both sites having a relatively lower diversity than the other two sites (Table 1).

Similarly, the composition of the BMI communities varied between the four sampling sites (Supplementary material Table S3), with values of the Bray-Curtis index ranging from 0.54 (PV*p vs.* TL*i*) to 0.96 (TL*p vs.* PV*i*). The TL*p* community differed the most from the other communities (Bray-Curtis index > 0.87) and was dominated by the gastropods Lymnaeidae and Planorobidae (Table S3). At PV*i*, there was the highest BMI density while the lowest was in TL*p* (Table 1). At PV*p* BMI community was relatively more diverse and richer than at the other sites (Table 1).

### Barbel age structure and condition

**Fig. 2:**
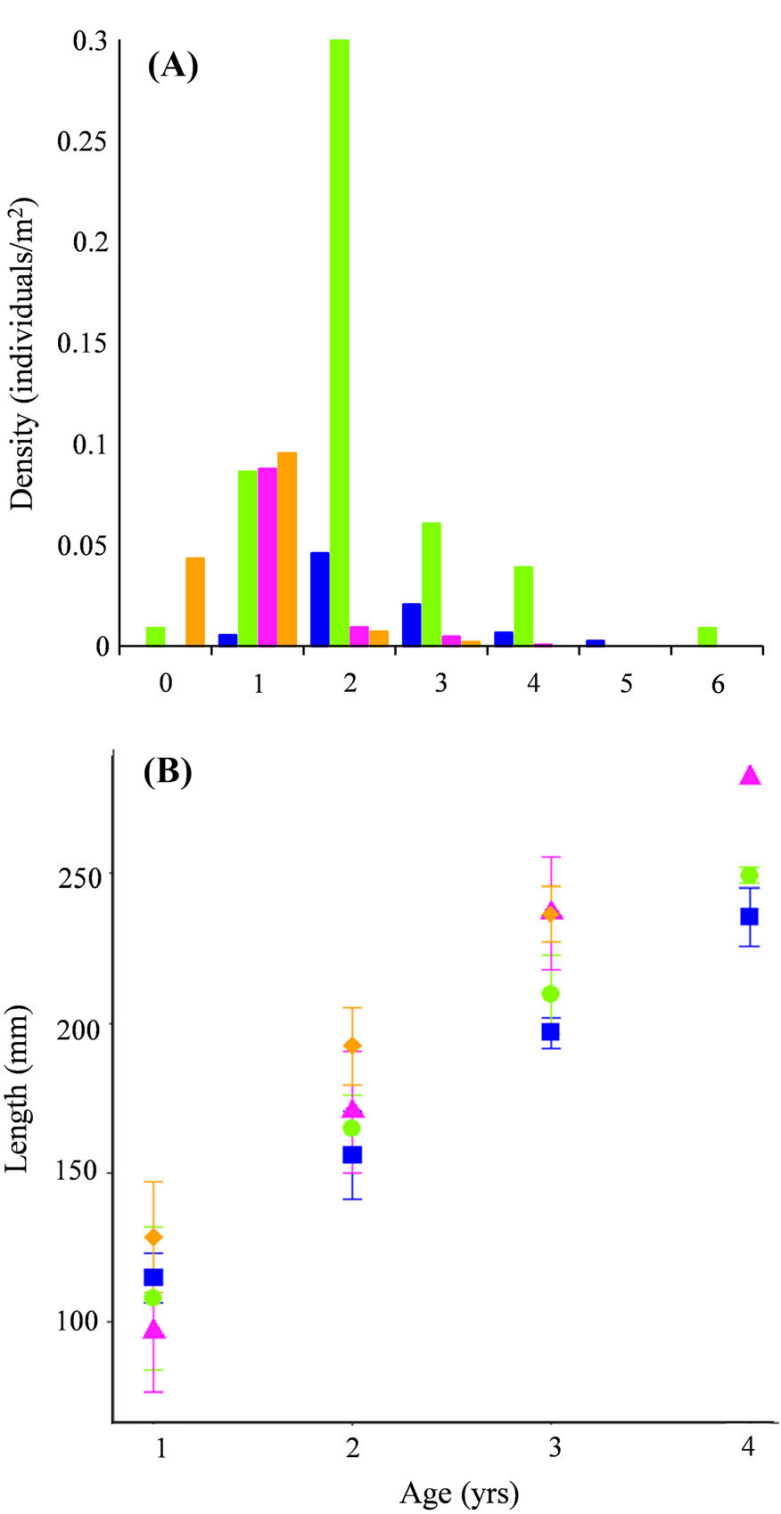
(**a**) Age class structure of barbel at each site where bars indicate density (individuals/m^2^) for each age class (0+ to 6+) of fish sampled at PV*p*, TL*p*, PV*i* and TL*i* respectively. (**b**) Mean total lengths (± standard deviations) of barbel of ages 1+ to 4+ sampled at PV*i* (pink triangles), TL*i* (orange diamonds), PV*p* (blue squares) and TL*p* (green circles) sites

Across the four populations, seven age classes (0 + to 6+) were present at fish lengths of 38 to 286 mm. Fish of 5+ and 6+ years were only present in the purebred barbel populations (TL*p* and PV*p*), with fish in the introgressed populations reaching a maximum age of 3+ (TL*i*) and 4+ (PV*i*) years (Fig. 2a). The most frequent age classes present were 1+ in hybrid populations and 2+ in purebred populations. As the length data were not homogeneously distributed in terms of number of individuals per age class, theoretical growth model calculations were performed on the mean total lengths, where data on the age 5+ and 6+ fish were not included as they were not present in all the populations. The model in which L_∞_ and t_0_ varied across the populations while K remained constant (K = 0.24 ± 0.03 standard error) was selected as the best-fitting model, indicating that the introgressed barbel (both at TL*i* and PV*i*) had significantly larger maximum theoretical lengths than purebreds (Table 2). MANOVA indicated significantly different length-at-age between sites for age classes from 1+ to 4+ (Pillai’s trace = 0.5; F_2,245_ = 31.67, *p* < 0.001), with hybrids having greater mean lengths at ages equal and/or greater than 2 years old (Fig. 2b).

**Table 2:** Mean ± standard error of length-weight relation (LWR) parameters with relative body index (BI), maximum theoretical lengths (L_∞_) and theoretical age at which the total length of the fish is equal to 0 (t_0_) calculated by the best-fitting von Bertalanffy (1938) model for introgressed (PV*i* and TL*i*) and purebred populations (PV*p* and TL*p*)

Population	N	LWR parameters					
		*a*	*b*	R^2^	BI	L_∞_	t_0_
PV*p*	41	0.015 ± 0.19	2.83 ± 0.07	0.98	0.01 ± 0.01	34.4 ± 1.8	-0.60 ± 0.10
PV*i*	72	0.016 ± 0.17	2.78 ± 0.07	0.96	0.01 ± 0.03	45.7 ± 3.1	0.03 ± 0.07
TL*p*	44	0.011 ± 0.10	2.99 ± 0.04	0.99	0.01 ± 0.01	35.9 ± 1.7	-0.50 ± 0.10
TL*i*	141	0.014 ± 0.07	2.80 ± 0.03	0.99	0.01 ± 0.02	41.1 ± 2.8	-0.55 ± 0.10

The rate of approach to L_∞_ remained constant (K = 0.24 ± 0.03) between the populations and it is not reported in the table. N = number of barbel analysed per population; *a* = intercept of the LWR regression curve, *b =* regression coefficient (slope), R^2^ = determination coefficient of the LWR regression curve

Length-weight relations (LWRs) varied significantly across the populations (ANCOVA: F_3, 293_ = 1430, *p* < 0.001) and within each population, LWR models were highly significant (R^2^ ≥ 0.96, *p* < 0.001; Table 2). Allometric negative growth (i.e. *b* < 3; *t*-test *p* < 0.05) was detected in all populations except for TL*p* (*b* = 3.0). Conversely, body condition indices were all around zero (Table 2) and did not vary between barbel populations (ANOVA F_3, 294_ = 1.35; *p* > 0.05).

### Barbel diet composition and dietary niche

**Fig. 3:**
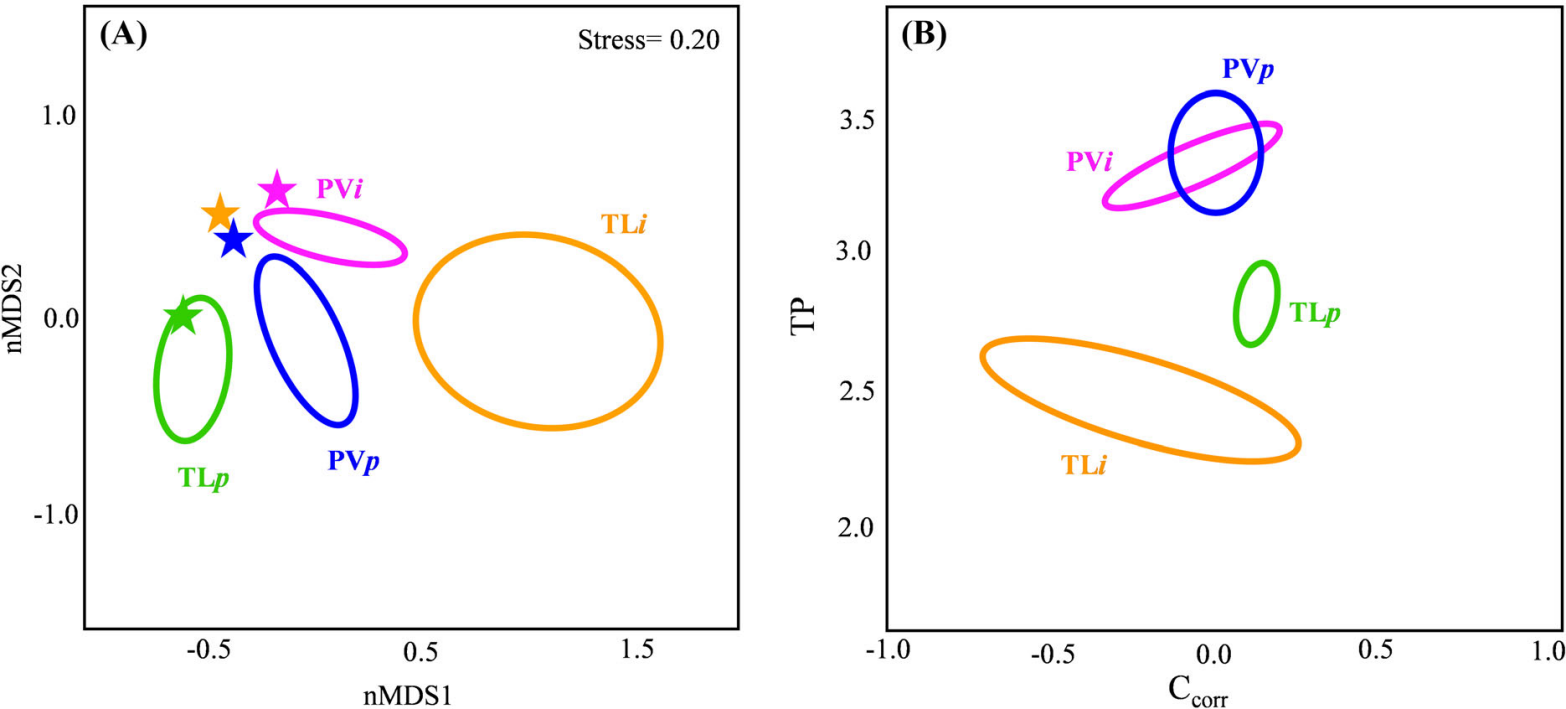
**a** Non-metric multidimensional scaling (nMDS) graph showing the dietary niches built as standard ellipses enclosing 40 % of the gut content data within each population and macroinvertebrate community composition at each site as indicated by colored star symbols. **b** Isotopic niches of each barbel population built on the corrected stable isotope data as maximum likelihood standard ellipse area (SEA) enclosing 40 % of the data for introgressed (PV*i* = pink and TL*i* = orange) and purebred (PV*p* = blue and TL*p* = green) barbel populations

The fish analysed for GCA were not significantly different in length across the rivers (ANOVA: F_3,77_ = 0.84; *p* > 0.05). The proportion of fish with empty guts (vacuity index, I %) ranged between 0 % (TL*p*) to 21 % (TL*i*) (Table 3), with mean gut fullness being the highest and the lowest in the same rivers respectively (ANOVA: F_3, 69_ = 14.86; *p* < 0.001). Prey accumulation curves were approaching the asymptote in all populations, suggesting the subsample of gut analysed was sufficient to represent the diet variability of barbel populations (Supplementary material Figure S1). The most frequent food items in barbel diets across all sites were aquatic insect larvae, particularly Chironomidae (Supplementary material Figure S2 and Table S4). Feeding strategy plots (Figure S2) indicated generalized feeding behaviour in all populations, with all barbel frequently consuming certain prey items (e.g. Chironomidae and Simuliidae), but with some differences in the contributions of others (e.g. Mollusca, terrestrial organisms and plants). However, an exception was in TL*i* and PV*i*, where there was some dietary specialization through some individuals feeding on fish (Figure S2). This resulted in considerable differences in diet composition among sites, with significant differences in the population trophic niches (PERMANOVA test: F_3, 69_ = 14.75, *R*^2^ = 0.40; *p* < 0.001). The widest trophic niche was in TL*i* and then PV*p* (as shown by 40 % ellipses in the nMDS analysis, Table 3; Fig. 3 A). All pairwise comparisons revealed significant differences in niche composition between the populations (*p*_adj_ < 0.01), with the highest overall average dissimilarity in the diet of TL*i* barbels (≥ 75.6 %; Table S4). The diet of hybrids in TL*i* lacked the items that were frequent and abundant in the diets of the other populations (e.g. Mollusca, Hydropsychidae and other Trichoptera), while consuming food categories (e.g. fish bones and plants) that were absent or infrequent in the other populations (Table S4 and Figure S1). The diet of barbel at TL*i* was also the most distant from the macroinvertebrate community present at the site of capture as shown by the nMDS analysis (Fig. 3a). Although there were significant differences in H between the diets of the barbel populations (ANOVA: F_3, 69_ = 11.76; *p* < 0.001), pairwise comparisons indicated these were only significant between TL*i* and the other sites (Tukey test, *p*_adj_ < 0.001) (Table 3).

**Table 3:** Mean vacuity index (I %) and mean percent gut fullness, Shannon-Wiener diversity index of diet (H) and dietary niche width estimated as 40 % nMDS ellipse area for purebred (TL*p* and PV*p*) and hybrid (TL*i* and PV*i*) barbel populations

Population	N	TL (range)	I %	Mean gut fullness (%)	H	Niche nMDS
PV*p*	20	155 (110–252)	10	62 ± 25	1.35 ± 0.50	0.26
PV*i*	20	160 (72–279)	10	60 ± 28	1.37 ± 0.34	0.14
TL*p*	22	180 (91–285)	0	85 ± 14	1.69 ± 0.24	0.21
TL*i*	19	159 (69–241)	21	32 ± 27	0.89 ± 0.52	0.90

Number of fish analysed for GCA per population (N), mean total length (TL) and relative range (mm) are also given

### Stable isotopes, barbel trophic position and isotopic niches

**Fig. 4:**
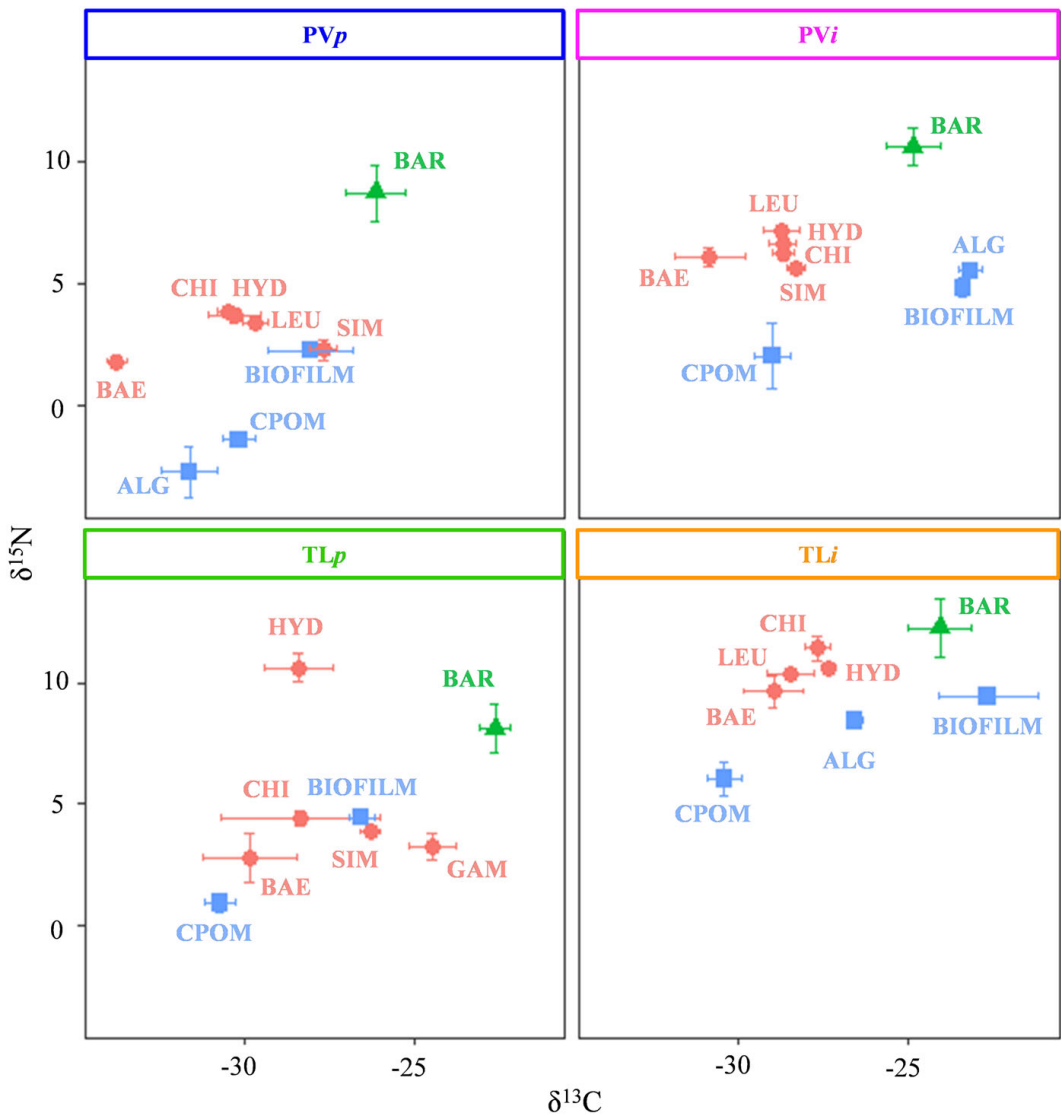
Stable isotope mean ratios with standard deviations (bars) of barbel (green triangles), macroinvertebrates (pink circles) and primary producers (blue squares) collected at four sites. BAR = barbel; macroinvertebrates: BAE = Baetidae, CHI = Chironomidae, HYD = Hydropsychidae, GAM = Gammaridae, LEU = Leuctridae, SIM = Simuliidae; primary producers: CPOM = coarse particulate organic matter, ALG = benthic algae. FPOM was omitted from the graph as it was particularly depleted in ^13^ C in almost all the sites (except PV*p*) compared to barbel and BMI and therefore not useful as baseline (cf. Table S1)

Across the four sites, δ^13^C BMI varied, with the carbon range being between 1.6 ‰ (TL*i*) and 6.1 ‰ (PV*p*) (Fig. 4; Table S1). FPOM was particularly ^13^C-enriched in all rivers except for TL*p* (Table S1). Values of δ^15^N were more similar for both BMI and primary producers between TL*p* and PV*i* (Fig. 4, Table S1), while there was an enrichment of ^15^N at TL*i*. In the barbel populations, there was no evidence of significant ontogenetic shifts in δ^15^N and δ^13^C (Supplementary material Table S5), except for fish in PV*p* where δ^13^C decreased as fish length increased (Table S5). There was no significant difference in the length of the fish analysed between rivers (F_3, 36_ = 0.15, *p* > 0.05).

A significantly lower trophic position (as indicated by posterior probability distributions) was detected for barbel in TL*i* compared to TP in the other populations (TP 2.4 vs. > 2.8; Table 4). No significant differences were found in C_corr_ between rivers (F_3, 36_ = 0.84, *p* > 0,05) and length was subsequently removed due to its non-significant effect (*p* > 0.05). The isotopic niche size of the barbel was significantly larger in TL*i* and smaller in TL*p* (as indicated by posterior distributions of the core isotopic niche as SEA_B_), with the niches being similarly sized in PV*p* and PV*i* (Table 4). In general, the positions of these niches in the isotopic space did not overlap except for PV*p* and PV*i* that shared 6 % of their core niches (Fig. 3b).

**Table 4:** Mean bulk stable isotope ratio ± standard deviation, Bayesian estimate of trophic position (TP) with relative 95 % credible intervals and corrected carbon values (C_corr_) ± standard deviation of each barbel population together with estimation of the isotopic niche breath calculated as Bayesian standard ellipse area SEA_B_ (95 % credible interval).

River	N	TL	δ^15^N_muscle_	TP	δ^13^C	C_corr_	SEA_B_
PV*p*	10	164 (103–242)	8.9 ± 1.1	3.4 (2.9–4.2)	-26.1 ± 0.9	0.0 ± 0.1	0.12 (0.06–0.23)
PV*i*	10	161 (98–239)	10.8 ± 0.7	3.0 (2.7–3.7)	-24.8 ± 0.8	-0.1 ± 0.3	0.11 (0.06–0.23)
TL*p*	10	172 (106–244)	8.3 ± 1.0	2.8 (2.2–3.4)	-22.6 ± 0.4	0.1 ± 0.1	0.04 (0.02–0.08)
TL*i*	10	168 (100–241)	12.4 ± 1.2	2.4 (2.1–2.8)	-24.0 ± 0.9	-0.2 ± 0.6	0.50 (0.25–0.98)

Number of samples analysed (N) and mean total length (TL) in mm and relative range (in brackets) are provided

## Discussion

The testing of the ecological and biological consequences of introgressive hybridisation of the endemic *B. tyberinus* and *B. plebejus* following *B. barbus* invasion revealed substantial changes in some biological traits of introgressed populations and an altered trophic ecology in one of the hybrid population. These results reveal important insight into the ecology of the hybridized progenies and highlight the potential impacts of hybridization at wider ecosystem scales.

The growth characteristics of the hybrids (*B. barbus* × *B. tyberinus* and *B. barbus* × *B. plebejus*), including their maximum theoretical lengths and lengths at age, were higher in the invaded populations than in the purebred populations, as per the prediction. These results are also similar to those from previous studies on *B. barbus* hybrid populations of central (Carosi et al. 2017) and northern Italy (Meraner et al. 2013), and suggest an element of hybrid vigour. Indeed, similar patterns of hybrid vigour have been recorded in other interbreeding fish species, such as *Cyprinodon pecosensis* Echelle & Echelle 1978 and its congener *C. variegatus* Lacepède 1803 (Rosenfield et al. 2004), the Japanese strain of *Cyprinus carpio* Linnaeus 1758 and its domestic exotic lineage (Matsuzaki et al. 2010), and *Abramis brama* (Linnaeus 1758) and *Rutilus rutilus* (Linnaeus 1758) (Toscano et al. 2010; Hayden et al. 2011). Moreover, hybrid vigour has been documented in a range of other animal and plant species (Burke and Arnold 2001; Pfennig et al. 2007; Hovick and Whitney 2014). The increased size of barbel hybrids may enhance recruitment through a higher number of eggs being spawned (Philippart and Berrebi 1990; Meraner et al. 2013; Gutmann Roberts et al. 2020) compared to the smaller native purebreds. This, along with the related vigour of the introgressed progenies, potentially helps to explain the rapid expansion of hybridization in invaded population by *B. barbus*. Alternatively, the larger size of alien barbel and its hybrids may play an active role in sexual selection, with larger females being more attractive to barbel males than the smaller native females (Meraner et al. 2013).

The second prediction concerned the differences in trophic ecology between hybrids and purebred barbel populations and were tested using a combination of gut contents (GCA) and stable isotope (SIA) analyses. These techniques are considered to be largely complementary (e.g. Nolan and Britton 2018) and are often used together in fish trophic studies (e.g. Locke et al. 2013; Hamidan et al. 2016), although they do reflect two different aspects of animals’ feeding ecology that can result in discordant outcomes (e.g. Pacioglu et al. 2019). Where GCA represents a dietary snapshot of an individual in real time, representing the prey consumed in the preceding hours, SI data integrate spatial and temporal dietary components over a period of days to months, depending on the actual tissue analysed (Vander Zanden et al. 2015). Here, scale material was used that, in *B. barbus*, has a relatively slow isotopic turnover rate compared to other tissues (Busst and Britton 2018), thus the temporal aspect of the diet being indicated was likely to be over 3 months. Despite these core methodological differences, the two methods were consistent in demonstrating some considerable differences in the diet composition and trophic niche of the TL*i* hybrid population compared to their reference parental population (TL*p*), and, conversely, only minor differences between the PV*i* hybrid population and its reference purebred population (PV*p*). The introgressed barbel of TL*i* differed to the other three populations studied in their relatively high proportions of small fishes and plants in their diet, which resulted in a relatively low trophic position. The diets of the other populations were all dominated by benthic macroinvertebrate prey. These differences were then reflected in their trophic niche size, with the hybrids in TL*i* having the broadest isotopic and trophic niches.

The relatively high proportion of prey fishes in the diet of the introgressed barbel of TL*i* aligns to some *B. barbus* populations having diets in which prey fishes are present, albeit usually in low frequencies (Piria et al. 2005; Gutmann Roberts et al. 2017). Recreational anglers also frequently capture larger individuals on baits comprising of high proportions of marine fishmeal, suggesting that fish prey are attractive to adult *B. barbus* (De Santis et al. 2019). The barbel of TL*i* were the only population here where small benthic fishes were detected at relatively high frequency in diet by GCA, despite considerable overlap in the body sizes present, and the presence of small benthic fishes in all sites. The diet of the TL*i* hybrid barbel had the lowest diversity (in terms of the Shannon-Wiener index) of all populations, but as these fish had both plant and fish material present then they actually had the widest trophic niche. Moreover, TL*i* site had the highest fish density and a lower macroinvertebrate density than PV*i* site, so these data suggest that the hybrids of TL*i* preyed upon smaller fish through the combination of their high availability and relatively lower availability of macroinvertebrate resources, a pattern that was not evident elsewhere (Supplementary material Table S2 and S3). We can thus speculate that the hybrids in TL*i* were primarily consuming common food resources in the site rather than preferentially selecting small fishes as dietary items. However, it highlights both their diet plasticity and a shift in their functional feeding guild (Noble et al. 2007) from primarily being insectivorous (Oberdoff et al. 1993; Piria et al. 2005; Corse et al. 2010) in other sites to being omnivorous in TL*i*. This functional shift is potentially important in the context of assessments of their ecological impact (Cucherousset and Olden 2011).

In terms of their age structure and growth, the hybrid populations were relatively similar, despite their trophic differences. These results are consistent with morphological analyses that were conducted on the same populations (Zaccara et al. 2020), where the TL*i* barbel showed a marked morphological differentiation from the purebred *B. tyberinus* in their body shape, whereas the hybrids of PV*i* resulted relatively similar to the morphology of the PV*p* barbel phenotype. In general, these hybrid populations were characterised by individuals with deeper bodies and larger caudal fins than in the purebred ones, which displayed body-shapes that were potentially better adapted to fast flowing waters and unsteady swimming mode (Meyers and Belk 2014). This then could partly explain the difference in the trophic ecology observed in this study, as the functional morphology of fish is an important driver of their diet (Klingenberg et al. 2003) through its influence on their foraging mode and efficiency of prey capture (Webb 1984).

Different habitat conditions and interspecific competition may have been responsible for some of the observed trophic shift of hybrids in TL*i* population. For example, differences in canopy cover, such those present between upstream (PV*p* and TL*p*) and downstream (PV*i* and TL*i*) sites, could have influenced the trophic strategy shift and niche broadening of fish in TL*i* (e.g. De Carvalho et al. 2019). Nevertheless, this was not apparent in the hybrid population of PV*i*, despite similar habitat conditions (i.e. canopy cover, river geomorphology, river width, altitude and distance from source; Table 1) thus suggesting that environmental parameters were unlikely to have been important in driving this trophic response. Interspecific competition with other fishes could also have influenced the trophic ecology of the studied hybrids. Indeed, the highest fish density was in TL*i*, in large part caused by the high density of the Arno goby *P. nigricans* (Table S2), an invertivore (Pompei et al. 2014). However, experimental studies have shown decreased trophic niche sizes in relation to interspecific competition in *B. barbus* and other cyprinids (Britton et al. 2019; De Santis et al. 2021), which contrasts with the findings of this study. Specialization and subsequent reduction in trophic niche breadth have also facilitated the coexistence between other omnivorous fishes when resources are limited (e.g. Neves et al. 2021). Therefore, by the effect of interspecific competition, we mightmight have expected the trophic niche of TL*i* population to be more restricted. Variation in the trophic ecology of different hybrid classes (i.e. differences in the extent of introgression) has been detected in hybrids between native Japanese *Cyprinus carpio* lineages and non-native strains (Matsuzaki et al. 2010). Invasion history (e.g. time since the first introduction), propagule pressure, habitat structure and disturbance are all factors that may contribute to the different genotypic composition of hybrid populations (Hayden et al. 2011; Corse et al. 2015). Thus, future studies may involve a higher number of populations representative of different habitat conditions and populations with different genotypic structure to verify to which extent the pattern observed in this study are driven by changes in the genotype and phenotype, versus those driven by differences in their environment, including in prey availability and interspecific competition (Corse et al. 2015).

In summary, the results here provide evidence of an ecological shift in one barbel population introgressed with the invader *B. barbus*. In this population, the morphological change in the hybrids was associated to their exploitation of different prey resources, although the extent to which this was also driven by differences in prey availability and habitat condition was unable to be tested. In both the introgressed populations studied, hybrids grew to considerably larger sizes and had larger lengths at age, suggesting a potential reproductive advantage compared to purebred fish. These results highlight, for the first time, that *B. barbus* invasion not only results in the introgression with congeners with consequent genetic pollution, but these introgressed fish may then interact quite differently within the receiving communities than their non-hybridised parents. Although the role of other factors could not be verified, these results support the hypothesis that invasive hybridisation is, potentially, a major driver of ecological change.

## Supplementary Information

Below is the link to the electronic supplementary material.

Supplementary file 1 (PDF 465 KB)

## Data Availability

All data produced in this study are available upon requests from the corresponding author;

## Code availability

All codes used in this study are available upon requests from the corresponding author.
