# Supplementary material for: Biological and trophic consequences of genetic introgression between endemic and invasive Barbus fishes
Source: Biol Invasions. 2021 May 26;23(11):3351–68. doi: 10.1007/s10530-021-02577-6 (PMC8149140; doi:10.1007/s10530-021-02577-6)
Supplement: Supplementary file 1 — (PDF 465 KB) [file 10530_2021_2577_MOESM1_ESM.pdf]

Supplementary information

Title: Biological and trophic consequences of genetic introgression between endemic and invasive *Barbus* fishes

Journal: Biological Invasions

Authors: Vanessa De Santis, Silvia Quadroni, Robert J. Britton, Antonella Carosi, Catherine Gutmann Roberts, Massimo Lorenzoni, Giuseppe Crosa, Serena Zaccara

Corresponding author: Vanessa De Santis- Department of Theoretical and Applied Sciences, University of Insubria, Varese, VA, Italy- v.desantis1@uninsubria.it

Supplementary Tables

Table S1 Mean ± standard deviation of δ^{15}N and δ^{13}C ratio of primary consumers (benthic macroinvertebrates, BMI) and primary producers (benthic algae, biofilm, coarse particulate organic matter (CPOM) and fine particulate organic matter (FPOM)) collected at the four sampling sites. Each category was represented by three replicates (N = 3).

Site	Group	Definition	δ^{13}C	δ^{15}N
PV*p*	BMI	Baetidae	-33.7 ± 0.3	1.8 ± 0.2
		Chironomidae	-30.5 ± 0.3	3.9 ± 0.3
		Hydropsychidae	-30.3 ± 0.8	3.7 ± 0.3
		Leuctridae	-29.7 ± 0.4	3.4 ± 0.2
		Simulidae	-27.7 ± 0.4	2.3 ± 0.4
	Primary producers	Benthic algae	-31.6 ± 0.8	-2.7 ± 1.0
		CPOM	-30.1 ± 0.5	-1.4 ± 0.3
		Biofilm	-28.1 ± 1.2	2.3 ± 0.1
		FPOM	-9.9 ± 0.4	1.9 ± 0.4
PV*i*	BMI	Baetidae	-30.8 ± 1.0	6.1 ± 0.4
		Chironomidae	-28.6 ± 0.3	6.2 ± 0.3
		Hydropsychidae	-28.7 ± 0.4	6.6 ± 0.2
		Leuctridae	-28.7 ± 0.5	7.2 ± 0.2
		Simulidae	-28.3 ± 0.3	5.6 ± 0.2
	Primary producers	Benthic algae	-23.1 ± 0.4	5.5 ± 0.3
		CPOM	-29.0 ± 0.5	2.0 ± 1.4
		Biofilm	-23.4 ± 0.2	4.8 ± 0.4
		FPOM	-8.4 ± 0.2	1.5 ± 1.5
TL*p*	BMI	Baetidae	-29.8 ± 1.4	2.8 ± 1.0
		Chironomidae	-28.3 ± 2.3	4.4 ± 0.3
		Gammaridae	-24.4 ± 0.7	3.3 ± 0.5
		Hydropsychidae	-28.4 ± 1.0	10.6 ± 0.6
		Simulidae	-26.3 ± 0.3	3.8 ± 0.1
	Primary producers	CPOM	-30.7 ± 0.4	1.0 ± 0.4
		Biofilm	-26.5 ± 0.4	4.4 ± 0.2
		FPOM	-23.1 ± 0.1	1.9 ± 0.3
TL*i*	BMI	Baetidae	-29.0 ± 0.9	9.6 ± 0.7
		Chironomidae	-27.7 ± 0.4	11.4 ± 0.5
		Hydropsychidae	-27.3 ± 0.2	10.6 ± 0.2
		Leuctridae	-28.4 ± 0.7	10.4 ± 0.2
	Primary producers	Benthic algae	-26.6 ± 0.2	8.4 ± 0.1
		CPOM	-30.4 ± 0.5	6.0 ± 0.7
		PP	-22.6 ± 1.5	9.4 ± 0.1
		FPOM	-10.9 ± 0.9	6.0 ± 0.8

Table S2 Fish species assemblage at each sampling site with relative density (individuals/m^2) of each species found. Superscript letters indicate exotic species ([e]) or translocated ones ([t]) (i.e. those native to PV and introduced in TL).

**Family**	**Species**	**Density (individuals/m^2)**			
Cobitidae	*Cobitis bilineata*		0.24		
Cottidae	*Cottus gobio*	1.84			
Cyprinidae	*B. plebejus*	0.08			
	B. barbus × B. plebejus[e]		0.10		
	B. barbus × B. tyberinus[e]				0.15
	B. tyberinus			0.51	
Gobiidae	*Padogobius bonelli*		0.38		
	Padogobius nigricans				7.00
Gobionidae	*Gobio gobio*[e]		1.08		
	Pseudorasbora parva[e]				0.02
Leuciscidae	*Alburnus alborella*[t]		0.04		0.01
	Leuciscus lucumonis			0.02	
	Protochondrostoma genei[t]		0.014		1.00
	Sarmarutilus rubilio	0.02	0.01	0.04	0.01
	Squalius squalus	0.06	0.11	0.01	0.06
	Telestes muticellus	1.40			
Salmonidae	*Oncorhynchus mykiss*[e]			0.21	
	Salmo trutta[e]	0.01			
Siluridae	*Silurus glanis*[e]				0.02

Table S3 Macroinvertebrate assemblage (family level) found at each sampling site with relative density (individuals/m^2) for each family found.

**Class/Order**	**Family**	**Density (individuals/m^2)**			
Amphipoda	Gammaridae		1	6	83
Bivalvia	Spheriidae				16
Coleoptera	Others	9	8	62	5
Diptera	Chironomidae	285	845	530	85
	Simuliidae	24	2267		17
	Others	24	2	11	6
Ephemeroptera	Baetidae	88	1415	74	10
	Caenidae		68	123	
	Ephemerellidae	86			
	Ephemeridae	2			4
	Heptageniidae	19	24	7	2
	Leptophlebiidae	92	9	29	
Gastropoda	Lymnaeidae	6	10		481
	Planorbidae				128
Odonata	Gomphidae	1	6		
Oligochaeta	Lumbricidae	8	5		2
Plecoptera	Leuctridae	354	168	64	1
Trichoptera	Hydropsychidae	64	805	300	
	Philopotamidae	27	4		
	Rhyacophilidae		25	6	6
	Others	5			2

Table S4 Mean volume proportions (V_m) of 12 macroinvertebrate taxa (when family is indicated, family name is preceded by order) and three broader categories composing the diet of barbel collected at the four sampling sites, and results of the SIMPER analysis of gut contents between each couple of sampling sites (overall average dissimilarity is reported within brackets). C % = percentage contribution of each item to the overall average dissimilarity.

	V_m				PV*p* vs. PV*i* (59.2)	TL*p* vs. TL*i* (81.0)	PV*p* vs. TL*p* (61.9)	PV*i* vs. TL*i* (75.6)	PV*p* vs. TL*i* (78.4)	PV*i* vs. TL*p* (65.9)
**Item**	**PV*p***	**PV*i***	**TL*p***	**TL*i***	**C%**					
Plecoptera Leuctridae	1.3	0	1.3	0.1	2.5	2.3	3.3	0.4	2.5	2.2
Ephemeroptera Baetidae	3.7	19.0	3.2	1.4	16.5	4.8	5.9	18.6	6.4	11.4
Other Ephemeroptera	3.5	1.5	1.6	1.3	6.2	3.5	5.0	4.9	6.2	3.8
Trichoptera Hydropsychidae	0.5	4.2	0.2	0	7.2	0.3	1.3	7.4	1.2	5.5
Other Trichoptera	1.6	0.9	6.2	3.3	4.2	7.1	7.9	5.7	5.7	7.0
Diptera Chironomidae	25.4	11.3	18.2	4.5	13.7	10.5	9.4	10.4	18.1	6.3
Diptera Limoniidae	9.5	0	1.5	0	12.1	2.4	8.8	0	11.7	2.5
Diptera Simuliidae	7.7	17.5	3.1	0.1	13.7	4.2	7.5	18.9	11.6	11.6
Other Diptera	0.1	0	4.5	0	0.3	6.6	6.8	0	0.3	6.6
Coleoptera Elmidae	1.9	0.2	2.6	0.5	4.4	4.2	4.7	1.0	4.4	4.2
Crustacea Gammaridae	0	0	7.7	0	0	8.7	9.2	0	0	8.8
Mollusca	4.6	2.7	25.4	0	8.0	18.1	15.6	5.2	7.0	15.6
Other macroinvertebrates	1.0	0.2	1.0	6.2	3.1	6.1	3.1	8.5	7.8	2.1
Fish bones	0	0.8	0	10.7	1.4	6.3	0	9.8	8.4	1.0
Terrestrial organisms	0.2	0.4	8.8	0.03	1.6	9.1	9.5	1.4	0.8	8.9
Plants	1.6	1.6	0	4.2	5.0	5.6	2.0	7.7	7.8	2.7

Table S5 Results of the regression models testing the relationships between fish length (mm) and stable isotope ratios ($\delta^{15}N$ and $\delta^{13}C$) tested as a proxy of ontogenetic effects on diets for purebred (PV*p* and TL*p*) and hybrid (PV*i* and TL*i*) barbel populations.

**Dependent variable**	**Population**	$F_{1,8}$	R^2	**p**
$\delta^{15}N$	PV*p*	0.01	0.01	> 0.05
	PV*i*	0.05	0.01	> 0.05
	TL*p*	1.64	0.17	> 0.05
	TL*i*	0.31	0.04	> 0.05
$\delta^{13}C$	PV*p*	30.13	0.79	< 0.001
	PV*i*	0.01	0.01	> 0.05
	TL*p*	2.62	0.24	> 0.05
	TL*i*	0.06	0.01	> 0.05

Supplementary figures

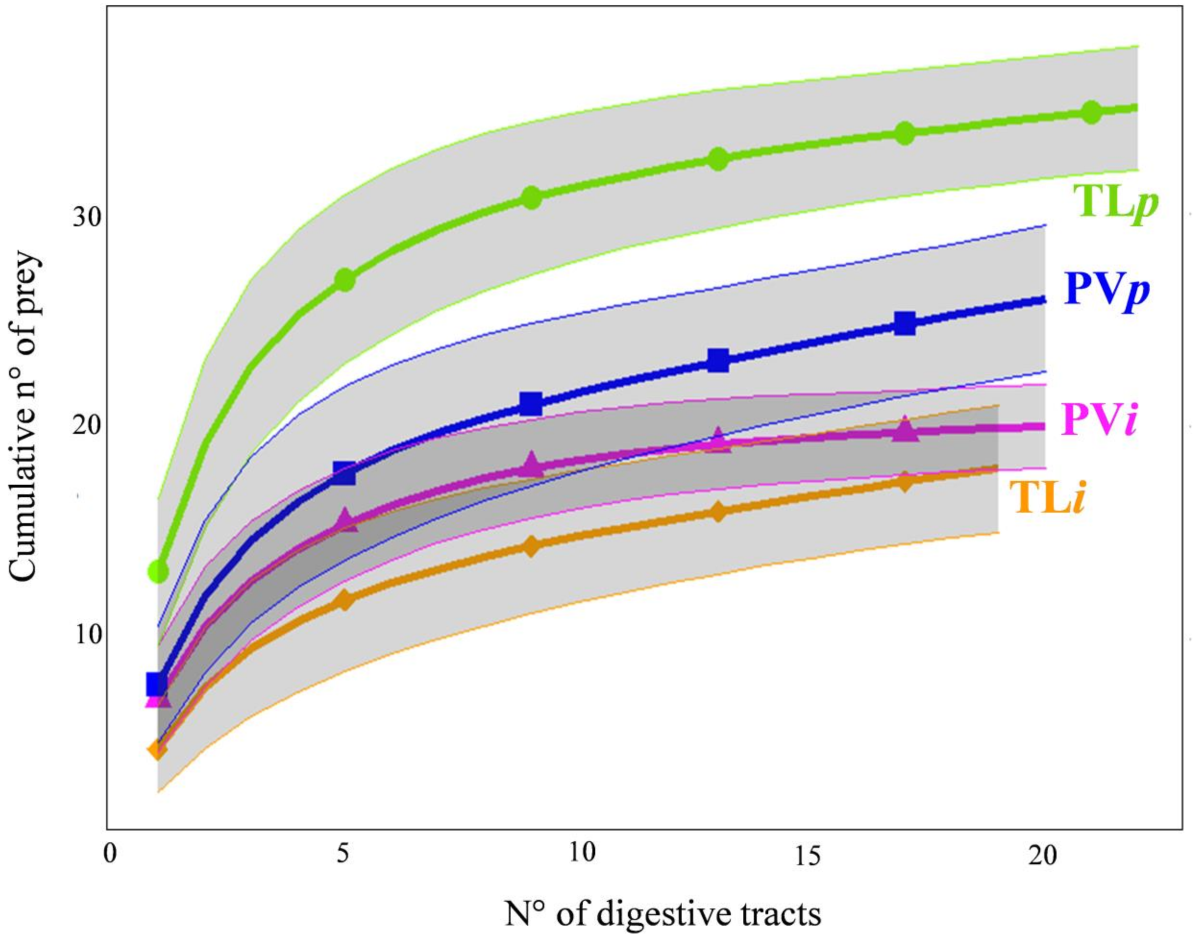

Figure S1 Prey accumulation curve of each barbel population indicating the cumulative number of food items found in the digestive tract of each individual fish sampled at PV*i* (pink triangles), TL*i* (orange diamonds), PV*p* (blue squares) and TL*p* (green circles) sites.

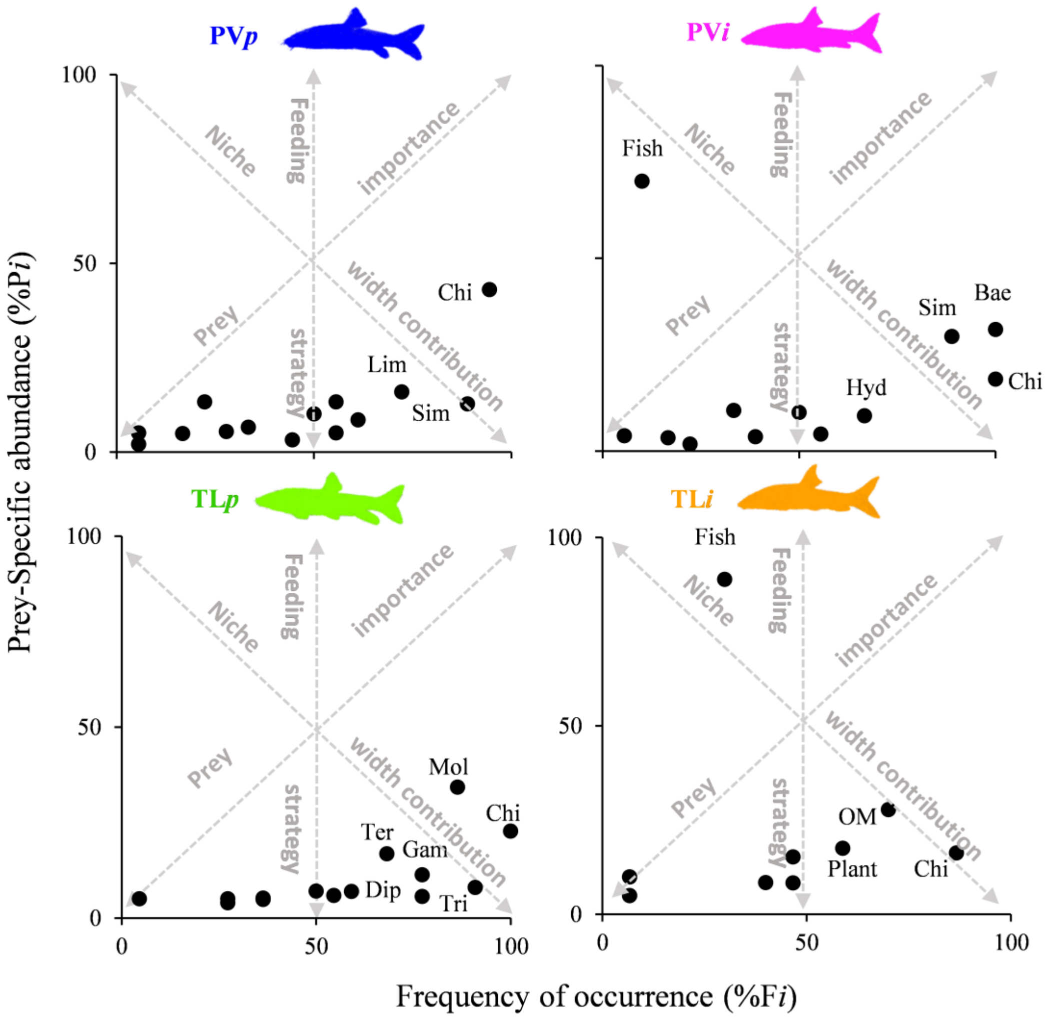

Figure S2 Feeding strategy plots (Costello et al., 1990; Amundsen et al., 1996) of each barbel population based on gut contents. Points indicate food items and name of the most frequent (%F*i* ≥ 59 %) or abundant items (%Pi ≥ 70%) are specified where: Fish = fish bones; Bae = Baetidae; Chi = Chironomid larvae; Dip = other Diptera; Gam = Gammaridae; Hyd = Hydropsychidae; Lim = Limoniidae; Mol = Mollusca; OA= other aquatic organisms; Plant = aquatic vegetation; Ter = terrestrial organisms; Tri= other Trichoptera. Prey importance (rare to dominant) increases along the diagonal from the bottom left to the upper right while feeding strategy changes along the vertical from the bottom up (generalist to specialist) and individual contribution to the trophic niche (i.e. between or within phenotypic contribution to the niche width) increases along the diagonal from the bottom right (high within phenotype contribution) to the upper left (high between phenotype contribution). See Amundsen et al., 1996 for further details on graph interpretation.
